# Supplementary material for: Native American genetic ancestry and pigmentation allele contributions to skin color in a Caribbean population
Source: eLife. 2023 Jun 9;12:e77514. doi: 10.7554/eLife.77514 (PMC10371226; doi:10.7554/eLife.77514)
Supplement: Supplementary file 1. [file elife-77514-supp1.docx]

**Supplementary File 1**

**Supplementary File 1A. Sample Demographics.**

| **Category** | **Entire sample**  (N=461) |
| --- | --- |
| **Sex**  male  female | 244  217 |
| **Age**  range  mean (SD)  median | 6 to 93  39 (21.5)  39 |
| **Paternal ancestry**  reported^a^  named  sampled^b^ | 432  193  49 |
| **Maternal ancestry**  reported^a^  named  sampled^c^ | 437  244  128 |

^a^ community-described ancestry collected.

^b,c^ values from reported genealogy; 75 fathers and 146 mothers as determined by genotyping.

**Supplementary File 1B: Summary of Kalinago ancestry from admixture analysis (n=458).** NAM = Native American, AFR = African, EUR = European, CSA = Central & South Asian, EAS = East Asian, OCE = Oceanian. At K=3, NAM, EAS, and OCE are not distinguishable.

| **K-value** | **AFR** | **NAM** | **EAS** | **OCE** | **EUR** | **CSA** |
| --- | --- | --- | --- | --- | --- | --- |
| 3 | 0.304 | 0.552 | | | 0.144 | |
| 4 | 0.318 | 0.549 | 0.011 | | 0.122 | |
| 5 | 0.318 | 0.548 | 0.011 | 0.002 | 0.121 | |
| 6 | 0.318 | 0.548 | 0.012 | 0.002 | 0.110 | 0.010 |

**Supplementary File 1C. Ancestry proportions estimated using different approaches.**

| **estimation approach** | **AMR** | **AFR** | **EUR** | **EAS** |
| --- | --- | --- | --- | --- |
| Admixture (subsets, K=4) | 0.549 | 0.318 | 0.122 | 0.011 |
| Admixture (two stage, K=4) | 0.541 | 0.316 | 0.126 | 0.016 |
| rfmix (4 clusters) | 0.553 | 0.313 | 0.125 | 0.009 |
| rfmix (3 clusters) | 0.557 | 0.326 | 0.117 | --- |

**Supplementary File 1D.i. Summary by locus of albinism candidates identified through exome sequencing.** Candidates are homozygous derived in one albino and heterozygous in one obligate carrier. No nonsense, frameshift, or splice variants was detected. Our initial attempt to identify the albinism variant in the Kalinago involved targeted genotyping of the albino individuals for 28 mutations previously observed^38,39,53,54^ in African or Native American albinos; these included the 2.7 kb exon 7 deletion in *OCA2* found at high frequency in some African populations. ­No mutation was detected using this approach.

| **OCA gene** | **Chromosome** | **Variants** | **Missense** |
| --- | --- | --- | --- |
| *OCA1 (TYR)* | 11 | 0 |  |
| *OCA2* | 15 | 5 | 2 |
| *OCA3 (TYRP1)* | 9 | 0 |  |
| *OCA4 (SLC45A2)* | 5 | 0 |  |
| *OCA5* | 4 | 6 | 0 |
| *OCA6 (SLC24A5)* | 15 | 0 |  |
| *OCA7 (LRMDA)* | 10 | 1 | 0 |

**Supplementary File 1D.ii. Characteristics of individual candidates identified through exome sequencing.**

| **Chr** | **rsID** | **Ref** | **Alt** | **f(AFR)^a^** | **Gene** | **Location/**  **Effect** |
| --- | --- | --- | --- | --- | --- | --- |
| 4 | rs3733437 | T | C | 0.126 | *EMCN* | intron |
| 4 | rs6826912 | T | G | 0.327 | *PPP3CA* | 3'UTR |
| 4 | rs463373 | T | C | 0.986 | *SLC39A8* | 3'UTR |
| 4 | rs439757 | C | A | 0.986 | *SLC39A8* | 3'UTR |
| 4 | rs223495 | A | G | 0.399 | *MANBA* | intron |
| 4 | rs3733632 | A | G | 0.819 | *TACR3* | 5'UTR |
| 10 | rs7911113 | A | G | 0.476 | *LRMDA* | intron |
| 15 | rs1800419 | A | G | 0.629 | *OCA2* | synonymous |
| 15 | rs1800401 | G | A | 0.126 | *OCA2* | *R305W* |
| 15 | rs797044784^b^ | CCAG | GACC | 0.002 | *OCA2* | *NW273KV* |
| 15 | rs73375883 | G | A | 0.203 | *OCA2* | intron |
| 15 | rs972334 | G | A | 0.217 | *OCA2* | intron |

^a^ Overall frequency for non-reference allele in seven 1KGP African populations.

^b^ 1KGP describes this variant as four consecutive SNPs rs549973474, rs569395077, rs538385900 and rs558126113.

**Supplementary File 1E: Effect sizes for covariates in linear regression model with 10 Principal Components.** Effect sizes are per allele for genomic variants (first allele only for albino variant). PC1 variance for included individuals (n=452) is 0.0045. P values adjusted using genomic control (applied to GWAS on the full variant set) are omitted if raw P value is above 0.05.

| **variable** | **Gene** | **standard** | **BETA** | **P_raw** | **Q (-log P)** | **alt1** |  | **BETA** | **P_raw** | **Q** | **Beta-ratio-std** |  | **alt2** | **BETA** | **P_raw** | **Q** | **beta-ratio-std** |
| --- | --- | --- | --- | --- | --- | --- | --- | --- | --- | --- | --- | --- | --- | --- | --- | --- | --- |
| rs7118677 | *GRM5/Tyr* | test | -2.419 | 0.0001392 | 3.86 | <omitted> |  |  |  |  |  |  | covariate | -2.403 | 9.32E-05 | 4.03 | 0.99 |
| rs1800404 | *OCA2* | test | -1.497 | 0.001446 | 2.84 | <omitted> |  |  |  |  |  |  | covariate | -1.256 | 0.005763 | 2.24 | 0.84 |
| rs885479 | *MC1R* | test | -1.31 | 0.008565 | 2.07 | <omitted> |  |  |  |  |  |  | covariate | -1.143 | 0.01684 | 1.77 | 0.87 |
| rs6917661 | *OPRM1* | test | -1.084 | 0.0117 | 1.93 | <omitted> |  |  |  |  |  |  | covariate | -1.004 | 0.01558 | 1.81 | 0.93 |
| rs2153271 | *BNC2* | test | -2.27 | 0.0002337 | 3.63 | <omitted> |  |  |  |  |  |  | covariate | -2.015 | 0.0007067 | 3.15 | 0.89 |
| *NW273KV* | *OCA2* | covariate | -7.744 | 9.00E-07 | 6.05 | covariate |  | -7.613 | 1.28E-06 | 5.89 | 0.98 |  | covariate | -8.61 | 1.54E-08 | 7.81 | 1.11 |
| *A111T* | *SLC24A5* | covariate | -5.757 | 2.45E-16 | 15.61 | covariate |  | -5.695 | 4.17E-16 | 15.38 | 0.99 |  | covariate | -5.76 | 1.29E-17 | 16.89 | 1.00 |
| *L374F* | *SLC45A2* | covariate | -4.415 | 3.90E-06 | 5.41 | covariate |  | -4.392 | 4.06E-06 | 5.39 | 0.99 |  | covariate | -3.667 | 6.87E-05 | 4.16 | 0.83 |
| PC1 |  | covariate | -62.14 | 5.73E-32 | 31.24 | covariate |  | -59.46 | 3.58E-28 | 27.45 | 0.96 |  | covariate | -60.52 | 1.42E-26 | 25.85 | 0.97 |
| PC2 |  | covariate | -0.0571 | 0.9904 | 0.00 | covariate |  | 0.7866 | 0.869 |  |  |  | covariate | 1.938 | 0.6903 | 0.16 |  |
| PC3 |  | covariate | 7.231 | 0.1208 | 0.92 | covariate |  | 7.891 | 0.09021 |  |  |  | covariate | 3.011 | 0.5015 | 0.30 |  |
| PC4 |  | covariate | -2.677 | 0.6025 | 0.22 | covariate |  | -2.242 | 0.6618 |  |  |  | covariate | -5.752 | 0.2477 | 0.61 |  |
| PC5 |  | covariate | -6.993 | 0.1447 | 0.84 | covariate |  | -6.714 | 0.1602 |  |  |  | covariate | -5.881 | 0.2115 | 0.67 |  |
| PC6 |  | covariate | 1.642 | 0.7392 | 0.13 | covariate |  | 1.309 | 0.79 |  |  |  | covariate | 4.788 | 0.3146 | 0.50 |  |
| PC7 |  | covariate | 7.277 | 0.1385 | 0.86 | covariate |  | 6.716 | 0.1707 |  |  |  | covariate | 8.29 | 0.08233 | 1.08 |  |
| PC8 |  | covariate | 3.267 | 0.5061 | 0.30 | covariate |  | 2.667 | 0.5867 |  |  |  | covariate | 8.227 | 0.08698 | 1.06 |  |
| PC9 |  | covariate | -17.27 | 0.001093 | 2.96 | covariate |  | -16.19 | 0.002222 |  |  |  | covariate | -17.86 | 0.0005783 | 3.24 |  |
| PC10 |  | covariate | 8.599 | 0.116 | 0.94 | covariate |  | 8.423 | 0.1224 |  |  |  | covariate | 6.41 | 0.2361 | 0.63 |  |
| SEX |  | covariate | 2.547 | 5.49E-05 | 4.26 | covariate |  | 2.515 | 6.45E-05 | 4.19 | 0.99 |  | covariate | 2.529 | 2.62E-05 | 4.58 | 0.99 |
| AGE |  | <omitted> |  |  |  | covariate |  | -0.02985 | 0.04596 |  |  |  | <omitted> |  |  |  |  |

This table compares three versions of analysis (linear regression only).

P values reported here (and Q = – log P) are not corrected for statistic inflation.

The last column for each non-standard case shows ratio of the effect size to that for the standard model, omitting PCs other than.

alt1 model adds age to standard analysis.

alt2 model adds five additional SNPs to standard analysis.

**Supplementary File 1F.** **Amplification conditions used for genotyping Kalinago samples for the selected alleles**.

| **Gene & Variant** | **Primer Sequence** | **PCR Annealing Temperature (°C)** |
| --- | --- | --- |
| *SLC24A5^A111T^*  rs1426654 | Fwd- CTCACCTACAAGCCCTCTGC  Rev- AATTGCAGATCCAAGGATGG | 55 |
| *SLC45A2^L374F^*  rs16891982 | Fwd- CCTGCTGGGACTCATCCATC  Rev- AGCAGAGTGCATGAGAAGGG | 55 |
| *OCA2^NW273KV^*  rs797044784 | Fwd- AGAGTCCCAGATGGTGTCTCA  Rev- AGGTCAGACTCCTTTAAACG | 53 |
| *OCA2^R305W^*  rs1800401 | Fwd- AGAGGGAGGTCCCCTAACTG  Rev- ATCTCAAGCCTCCCTGACTG | 53 |
| *MFSD12^Y182H^*  rs2240751 | Fwd- CCCAGGTGGAATAGCAGTGAG  Rev- AGTGGTTGGAATCACCTGTCA | 61 |
