# Supplementary material for: Native American genetic ancestry and pigmentation allele contributions to skin color in a Caribbean population
Source: eLife. 2023 Jun 9;12:e77514. doi: 10.7554/eLife.77514 (PMC10371226; doi:10.7554/eLife.77514)
Supplement: Supplementary file 2. [file elife-77514-supp2.docx]

**Supplementary File 2: Effect sizes of previously reported variants in Kalinago samples.**

| **CHR** | **pos (b37)** | **SNP** | **REF** | **ALT** | **gene** | **location** | **CADD_PHRED** | **Polyphen (main)** | **SIFT (main)** | **Freq** | **GT source** | **AR2** |  | **BETA_a** | **P_a_raw** | **P_a_adj** |  | **BETA_b** | **P_b** |  | **BETA_c** | **P_c** |  | **BETA_d** | **P_d** |  | **reference(s)** |
| --- | --- | --- | --- | --- | --- | --- | --- | --- | --- | --- | --- | --- | --- | --- | --- | --- | --- | --- | --- | --- | --- | --- | --- | --- | --- | --- | --- |
| 2 | 25329016 | rs12233134 | C | T | EFR3B near POMC | intronic | 0.348 | - | - | 0.473 | IMP | 1 |  | 0.61 | 0.197 | 0.2653 |  | -0.06 | 0.910365 |  | 0.15 | 0.771545 |  | 0.23 | 0.657335 |  | Quillen 2012 |
| 6 | 457748 | rs4959270 | C | A | LOC105374875 near IRF4 | intronic | 1.041 | - | - | 0.329 | GT | 1 |  | 0.07 | 0.8796 | 0.8959 |  | -0.14 | 0.783747 |  | -0.08 | 0.880465 |  | -0.07 | 0.896826 |  | Sulem 2007 |
| 6 | 466033 | rs1540771 | C | T | LOC105374875 near IRF4 | intronic | 0.95 | - | - | 0.305 | GT | 1 |  | -0.02 | 0.9703 | 0.9744 |  | -0.15 | 0.765123 |  | -0.08 | 0.875216 |  | -0.09 | 0.853243 |  | Sulem 2007 |
| 6 | 154663568 | rs2333857 | A | G | IPCEF1 near OPRM1 | intronic or upstream | 3.27 | - | - | 0.813 | IMP | 1 |  | 1.33 | 0.02932 | 0.05976 |  | 0.78 | 0.225651 |  | 1.16 | 0.0719104 |  | 1.22 | 0.059139 |  | Quillen 2012 |
| 6 | 154721557 | rs6917661 | C | T | CNKSR3 near OPRM1 | 3'UTR or downstream | 1.824 | - | - | 0.584 | GT | 1 |  | 1.08 | 0.0117 | 0.02938 |  | 0.61 | 0.20166 |  | 0.72 | 0.133223 |  | 0.76 | 0.111546 |  | Quillen 2012 |
| 7 | 55109177 | rs12668421 | A | T | EGFR | intronic | 0.212 | - | - | 0.494 | IMP | 0.98 |  | -0.16 | 0.7473 | 0.7809 |  | -0.39 | 0.46139 |  | -0.14 | 0.787191 |  | -0.15 | 0.780554 |  | Quillen 2012 |
| 7 | 55156071 | rs11238349 | G | A | EGFR | intronic | 0.431 | - | - | 0.393 | IMP | 1 |  | 0.34 | 0.48 | 0.542 |  | -0.65 | 0.22091 |  | -0.37 | 0.479127 |  | -0.34 | 0.523937 |  | Quillen 2012 |
| 7 | 55454267 | rs4948023 | G | A | LANCL2 near EGFR | intronic | 4.667 | - | - | 0.684 | IMP | 1 |  | 0.16 | 0.7641 | 0.7956 |  | -0.22 | 0.696131 |  | 0.00 | 0.996604 |  | 0.00 | 0.994267 |  | Quillen 2012 |
| 9 | 12682663 | rs10809826 | C | G | TYRP1 | upstream | 1.738 | - | - | 0.117 | IMP | 0.96 |  | -0.28 | 0.6803 | 0.7221 |  | -0.55 | 0.472775 |  | -0.65 | 0.392833 |  | -0.73 | 0.332409 |  | Adhikari 2019 |
| 9 | 16864521 | rs2153271 | C | T | BNC2 | intronic | 20.5 | - | - | 0.152 | GT | 1 |  | -2.27 | 0.0002337 | 0.001459 |  | -2.29 | 0.00110657 |  | -2.25 | 0.00120438 |  | -2.37 | 0.00065015 |  | Ju & Mathieson 2021 |
| 10 | 119564143 | rs11198112 | C | T | near EMX2 | intergenic | 17.79 |  |  | 0.187 | GT | 1 |  | 0.33 | 0.5664 | 0.6206 |  | 1.10 | 0.067385 |  | 0.99 | 0.100263 |  | 0.96 | 0.11598 |  | Adhikari 2019 |
| 11 | 88511524 | rs7118677 | G | T | GRM5 near TYR | intronic | 2.034 | - | - | 0.144 | IMP | 1 |  | -2.42 | 0.0001392 | 0.000982 |  | -1.62 | 0.0152453 |  | -1.99 | 0.00286815 |  | -2.10 | 0.00151048 |  | Adhikari 2019 |
| 11 | 88911696 | rs1042602 | C | A | TYR | S192Y | 23.8 | probably_damaging(0.974) | deleterious(0.01) | 0.072 | IMP | 0.74 |  | -2.90 | 0.0003701 | 0.002074 |  | -2.16 | 0.0119931 |  | -2.43 | 0.00469847 |  | -2.56 | 0.00273999 |  | Stokowski 2007 |
| 11 | 89011046 | rs1393350 | G | A | TYR | intronic | 1.555 | - | - | 0.019 | GT | 1 |  | -3.51 | 0.01947 | 0.04354 |  | -2.90 | 0.0869741 |  | -3.38 | 0.042737 |  | -3.57 | 0.0261186 |  | Liu 2015 |
| 11 | 89017961 | rs1126809 | G | A | TYR | R402Q | 27.2 | probably_damaging(0.994) | deleterious(0.03) | 0.019 | IMP | 0.97 |  | -3.51 | 0.01947 | 0.04354 |  | -2.90 | 0.0869741 |  | -3.38 | 0.042737 |  | -3.57 | 0.0261186 |  | Adhikari 2019; Ju & Mathieson 2021 |
| 12 | 89299746 | rs642742 | C | T | KITLG | upstream | 14.92 | - | - | 0.569 | GT | 1 |  | -0.34 | 0.4757 | 0.538 |  | -0.20 | 0.708049 |  | -0.30 | 0.568757 |  | -0.28 | 0.602548 |  | Sturm 2009 |
| 12 | 89328335 | rs12821256 | T | C | KITLG | upstream | 15.74 | - | - | 0.015 | GT | 1 |  | 1.20 | 0.5327 | 0.5902 |  | -1.36 | 0.524421 |  | -1.08 | 0.608744 |  | -0.95 | 0.648883 |  | Ju & Mathieson 2021 |
| 14 | 92773663 | rs12896399 | G | T | LOC105370627 near SLC24A4 | intronic | 0.043 | - | - | 0.054 | GT | 1 |  | -0.16 | 0.8692 | 0.887 |  | -0.96 | 0.352238 |  | -0.89 | 0.380027 |  | -0.98 | 0.337781 |  | Sulem 2007 |
| 15 | 28197037 | rs1800414 | T | C | OCA2 | H615R | 23.3 | benign(0.133) | deleterious(0) | 0.070 | IMP | 0.26 |  | 0.48 | 0.6116 | 0.6612 |  | -0.01 | 0.989592 |  | 0.07 | 0.939843 |  | 0.08 | 0.938174 |  | Edwards 2010 |
| 15 | 28213850 | rs4778219 | C | T | OCA2 | intronic | 1.527 | - | - | 0.316 | GT | 1 |  | -0.53 | 0.3074 | 0.3782 |  | -0.89 | 0.0952479 |  | -0.93 | 0.083945 |  | -0.90 | 0.0977717 |  | Adhikari 2019 |
| 15 | 28235773 | rs1800404 | C | T | OCA2 | synonymous coding | 0.321 | - | - | 0.488 | GT | 1 |  | -1.50 | 0.001446 | 0.005889 |  | -1.47 | 0.00324612 |  | -1.30 | 0.00924527 |  | -1.37 | 0.00639755 |  | Crawford 2017; Adhikari 2019i |
| 15 | 28260053 | rs1800401 | G | A | OCA2 | R305W | 22.7 | benign(0.245) | deleterious(0.03) | 0.068 | GT |  |  | 0.03 | 0.9788 | 0.9814 |  |  |  |  |  |  |  |  |  |  | --- |
| 15 | 28344238 | rs7495174 | A | G | OCA2 | intronic | 7.622 | - | - | 0.087 | GT | 1 |  | 1.64 | 0.0326 | 0.0649 |  | 1.57 | 0.0663265 |  | 1.60 | 0.0580815 |  | 1.60 | 0.0567887 |  | Han 2008 |
| 15 | 28365618 | rs12913832 | A | G | HERC2 near OCA2 | intronic | 15.8 | - | - | 0.074 | GT | 1 |  | -1.86 | 0.03926 | 0.07497 |  | -1.48 | 0.112546 |  | -1.53 | 0.100514 |  | -1.57 | 0.0919142 |  | Liu 2015; Adhikari 2019 |
| 15 | 28380518 | rs4778249 | T | A | HERC2 near OCA2 | intronic | 0.649 | - | - | 0.790 | IMP | 1 |  | -2.23 | 0.0002214 | 0.0014 |  | -2.43 | 0.00041828 |  | -2.14 | 0.00162008 |  | -2.12 | 0.0016832 |  | Adhikari 2019 |
| 15 | 28530182 | rs1667394 | C | T | HERC2 near OCA2 | intronic | 1.111 | - | - | 0.452 | GT | 1 |  | -1.42 | 0.002039 | 0.007666 |  | -1.56 | 0.00151414 |  | -1.41 | 0.0038335 |  | -1.43 | 0.00369895 |  | Sulem 2007 |
| 16 | 89986117 | rs1805007 | C | T | MC1R | R151C | 25.2 | probably_damaging(0.996) | deleterious(0.02) | 0.016 | GT | 1 |  | 1.32 | 0.4775 | 0.5397 |  | 0.67 | 0.712981 |  | 0.93 | 0.613105 |  | 0.86 | 0.64501 |  | Ju & Mathieson 2021 |
| 16 | 89986154 | rs885479 | G | A | MC1R | R163Q | 10.89 | benign(0.013) | tolerated(0.3) | 0.461 | IMP | 0.92 |  | -1.31 | 0.008565 | 0.0231 |  | -1.60 | 0.00241496 |  | -1.46 | 0.00572561 |  | -1.48 | 0.00546969 |  | Liu 2015 |
| 19 | 3548231 | rs2240751 | A | G | MFSD12 | Y182H | 27.4 | probably_damaging(0.999) | deleterious(0) | 0.031 | GT |  |  | -3.03 | 0.03735 | 0.0723 |  | -1.60 | 0.281792 |  | -1.46 | 0.33655 |  | -1.57 | 0.3027 |  | Adhikari 2019 |
| 20 | 3625436 | rs562926 | C | T | ATRN | intronic or downstream | 4.601 | - | - | 0.402 | GT | 1 |  | 0.85 | 0.08705 | 0.1395 |  | -0.12 | 0.821567 |  | 0.18 | 0.730685 |  | 0.28 | 0.595369 |  | Quillen 2012 |
| 20 | 32856998 | rs6058017 | A | G | ASIP/AHCY | 3'UTR/intron | 7.639 | - | - | 0.342 | IMP | 0.95 |  | -0.90 | 0.07274 | 0.1212 |  | -0.85 | 0.126901 |  | -1.04 | 0.0606301 |  | -1.04 | 0.0609305 |  | Stokowski 2007 |

key

a = linear regression, 10 PCs

b = LMM with 0 PCs, std GRM

c = LMM with 10 PCs, std GRM

d =LMM with 10 PCs, reap GRM
