# Supplementary material for: Native American genetic ancestry and pigmentation allele contributions to skin color in a Caribbean population
Source: eLife. 2023 Jun 9;12:e77514. doi: 10.7554/eLife.77514 (PMC10371226; doi:10.7554/eLife.77514)
Supplement: Supplementary file 3. [file elife-77514-supp3.docx]

**Supplementary File 3: Top novel variants that may contribute towards skin pigmentation from our GWAS analysis.** While the lowest p-values from the LMM-based methods meet the conventional criterion of 5e-08 for genome wide significance, the low observed minor allele frequencies (<2%) are inconsistent with what would be expected for variants responsible for pigmentation differences between the African and Native American populations.

| **CHR** | **pos (b37)** | **SNP** | **REF** | **ALT** | **gene** | **location** | **CADD_PHRED** | **Freq** | **GT source** | **AR2** |  | **BETA_a** | **P_a_raw** | **P_a_adj** |  | **BETA_b** | **P_b** |  | **BETA_c** | **P_c** |  | **BETA_d** | **P_d** |
| --- | --- | --- | --- | --- | --- | --- | --- | --- | --- | --- | --- | --- | --- | --- | --- | --- | --- | --- | --- | --- | --- | --- | --- |
| 1 | 114560208 | rs113236485 | A | G | near SYT6 | intergenic | 1.323 | 0.014 | IMP | 0.91 |  | 11.96 | 1.01E-08 | 6.71E-07 |  | 12.24 | 1.57E-07 |  | 12.91 | 4.67E-08 |  | 12.96 | 2.17E-08 |
| 1 | 114576742 | rs145925324 | G | A | near SYT6 | intergenic | 0.648 | 0.013 | IMP | 1 |  | 12.50 | 1.09E-08 | 7.12E-07 |  | 12.56 | 1.55E-07 |  | 13.39 | 3.85E-08 |  | 13.49 | 1.81E-08 |
| 1 | 114581335 | rs141998140 | G | T | near SYT6 | intergenic | 2.099 | 0.013 | IMP | 1 |  | 12.50 | 1.09E-08 | 7.12E-07 |  | 12.56 | 1.55E-07 |  | 13.39 | 3.85E-08 |  | 13.49 | 1.81E-08 |
| 1 | 114582335 | rs187318390 | C | T | near SYT6 | intergenic | 1.165 | 0.013 | IMP | 1 |  | 12.50 | 1.09E-08 | 7.12E-07 |  | 12.56 | 1.55E-07 |  | 13.39 | 3.85E-08 |  | 13.49 | 1.81E-08 |
| 1 | 114586703 | rs149623066 | A | G | near SYT6 | intergenic | 0.052 | 0.013 | IMP | 1 |  | 12.50 | 1.09E-08 | 7.12E-07 |  | 12.56 | 1.55E-07 |  | 13.39 | 3.85E-08 |  | 13.49 | 1.81E-08 |
| 1 | 114595150 | rs78273840 | C | T | near SYT6 | intergenic | 1.805 | 0.017 | IMP | 0.78 |  | 8.87 | 3.13E-06 | 5.39E-05 |  | 10.59 | 5.74E-07 |  | 11.11 | 1.98E-07 |  | 11.25 | 9.38E-08 |
| 1 | 114611620 | rs116218201 | T | G | near SYT6 | intergenic | 2.199 | 0.012 | IMP | 0.83 |  | 14.09 | 1.12E-09 | 1.24E-07 |  | 14.18 | 3.11E-08 |  | 15.52 | 3.16E-09 |  | 15.69 | 1.23E-09 |
| 1 | 114612965 | rs116746819 | G | A | near SYT6 | intergenic | 0.351 | 0.012 | IMP | 0.84 |  | 14.09 | 1.12E-09 | 1.24E-07 |  | 14.18 | 3.11E-08 |  | 15.52 | 3.16E-09 |  | 15.69 | 1.23E-09 |
| 1 | 114614230 | rs549514340 | T | C | near SYT6 | intergenic | 3.308 | 0.012 | IMP | 0.84 |  | 14.09 | 1.12E-09 | 1.24E-07 |  | 14.18 | 3.11E-08 |  | 15.52 | 3.16E-09 |  | 15.69 | 1.23E-09 |
| 1 | 114619521 | rs115102845 | C | G | SYT6 | downstream | 0.253 | 0.012 | IMP | 0.86 |  | 14.09 | 1.12E-09 | 1.24E-07 |  | 14.18 | 3.11E-08 |  | 15.52 | 3.16E-09 |  | 15.69 | 1.23E-09 |
| 1 | 114620469 | rs182159269 | G | A | SYT6 | downstream | 0.225 | 0.012 | IMP | 0.85 |  | 14.09 | 1.12E-09 | 1.24E-07 |  | 14.18 | 3.11E-08 |  | 15.52 | 3.16E-09 |  | 15.69 | 1.23E-09 |
| 1 | 114620940 | rs183827287 | C | T | SYT6 | downstream | 0.12 | 0.012 | IMP | 0.92 |  | 14.09 | 1.12E-09 | 1.24E-07 |  | 14.18 | 3.11E-08 |  | 15.52 | 3.16E-09 |  | 15.69 | 1.23E-09 |
| 1 | 114622601 | rs141251595 | G | A | SYT6 | downstream | 2.003 | 0.012 | IMP | 0.93 |  | 14.09 | 1.12E-09 | 1.24E-07 |  | 14.18 | 3.11E-08 |  | 15.52 | 3.16E-09 |  | 15.69 | 1.23E-09 |
| 1 | 114624033 | rs186173861 | T | C | SYT6 | downstream | 2.87 | 0.012 | IMP | 0.99 |  | 14.09 | 1.12E-09 | 1.24E-07 |  | 14.18 | 3.11E-08 |  | 15.52 | 3.16E-09 |  | 15.69 | 1.23E-09 |
| 1 | 114624337 | rs181299762 | C | T | SYT6 | downstream | 1.726 | 0.012 | IMP | 0.99 |  | 14.09 | 1.12E-09 | 1.24E-07 |  | 14.18 | 3.11E-08 |  | 15.52 | 3.16E-09 |  | 15.69 | 1.23E-09 |
| 1 | 114625992 | rs111898196 | T | C | SYT6 | downstream | 14.83 | 0.012 | IMP | 0.98 |  | 14.09 | 1.12E-09 | 1.24E-07 |  | 14.18 | 3.11E-08 |  | 15.52 | 3.16E-09 |  | 15.69 | 1.23E-09 |
| 1 | 114626277 | rs546818700 | T | A | SYT6 | downstream | 0.549 | 0.012 | IMP | 0.98 |  | 14.09 | 1.12E-09 | 1.24E-07 |  | 14.18 | 3.11E-08 |  | 15.52 | 3.16E-09 |  | 15.69 | 1.23E-09 |
| 1 | 114626295 | rs112626676 | C | T | SYT6 | downstream | 6.822 | 0.012 | IMP | 0.98 |  | 14.09 | 1.12E-09 | 1.24E-07 |  | 14.18 | 3.11E-08 |  | 15.52 | 3.16E-09 |  | 15.69 | 1.23E-09 |
| 1 | 114656630 | rs185469828 | C | T | SYT6 | intronic | 3.735 | 0.011 | IMP | 1 |  | 13.32 | 7.49E-08 | 3.12E-06 |  | 13.55 | 1.61E-06 |  | 15.25 | 1.58E-07 |  | 15.44 | 6.93E-08 |
| 1 | 114656848 | rs79537167 | G | T | SYT6 | intronic | 0.359 | 0.011 | IMP | 1 |  | 13.32 | 7.49E-08 | 3.12E-06 |  | 13.55 | 1.61E-06 |  | 15.25 | 1.58E-07 |  | 15.44 | 6.93E-08 |
| 3 | 124343256 | rs676091 | T | G | KALRN | intronic | 3.169 | 0.017 | IMP | 0.99 |  | 9.59 | 3.60E-08 | 1.78E-06 |  | 9.73 | 8.49E-08 |  | 9.02 | 6.00E-07 |  | 9.21 | 2.08E-07 |
| 4 | 65768709 | rs6816819 | T | G | LOC107986284 | intronic | 5.827 | 0.013 | GT | 1 |  | 11.99 | 6.11E-10 | 7.84E-08 |  | 10.96 | 1.14E-07 |  | 10.30 | 5.30E-07 |  | 10.50 | 1.66E-07 |

key

a = linear regression, 10 PCs

b = LMM with 0 PCs, std GRM

c = LMM with 10 PCs, std GRM

d = LMM with 10 PCs, reap GRM

ad j= based on lambda, inflation factor

beta = effect size
